# Work engagement: the key driver in transforming organizational commitment into enhanced work performance among midwives in Ghana - a structural equation modelling approach

**DOI:** 10.1186/s12913-025-13062-4

**Published:** 2025-07-01

**Authors:** Iddrisu Sisala Mohammed, Abubakari Wuni, Brenda Abena Nyarko, Mudasir Mohammed Ibrahim, Letitia Chanayireh

**Affiliations:** 1 Department of Nursing, Nurses’ and Midwives’ Training College, Tamale, Ghana; 2 College of Nursing, University of Kentucky, Lexington, USA; 3 Eliane Marieb College of Nursing, University of Massachusetts, Amherst, USA; 4 Department of Midwifery and Women’s Health, School of Nursing and Midwifery, University for Development Studies, Tamale, Ghana

**Keywords:** Organizational commitment, Work engagement, Work performance, Midwives

## Abstract

**Background:**

Midwives play a pivotal role in maternal and child health systems, yet their performance is profoundly shaped by organizational commitment and work engagement.

**Aim:**

This study examined the mediating effect of work engagement on the relationship between organizational commitment and work performance among midwives in Ghana.

**Method:**

An analytical cross-sectional design was employed in this study. Data were collected from 254 midwives using validated scales to assess organizational commitment, work engagement, and work performance. Analyses were conducted using SPSS Statistics and AMOS for structural equation modeling (SEM).

**Results:**

Most participants were female (96.9%) and aged 30–39 years (54.3%). Organizational commitment was positively correlated with work engagement (*r* = 0.125, *p* < 0.05) and work performance (*r* = 0.166, *p* < 0.05). Work engagement also showed a strong positive correlation with work performance (*r* = 0.662, *p* < 0.05). Mediation analysis confirmed that work engagement fully mediated the relationship between organizational commitment and work performance (β = 0.078, *SE* = 0.061, *p* < 0.001).

**Conclusion:**

The results of the study highlight the vital role of work engagement as a positive and significant mediator between organizational commitment and work performance among midwives. Investing in strategies that boost midwives’ commitment and engagement is essential for strengthening maternal healthcare quality and workforce sustainability.

## Introduction

Healthcare systems worldwide rely heavily on the competence, motivation, and performance of frontline healthcare workers [1]. Prolonged exposure to occupational stress, however, has been shown to adversely affect these professionals’ health, work performance, and overall quality of patient care [2]. Midwives, in particular, frequently work under demanding conditions marked by resource constraints, excessive workloads, limited professional development opportunities, and inadequate institutional support [3]. Such challenges not only hinder their effectiveness but also jeopardize the quality of care provided to mothers and newborns [4].

Midwifery is an inherently emotionally demanding profession. Supporting women and their families through complex social and clinical situations often exposes midwives to profound emotional burdens, including anxiety, fear, and grief [5]. These stressors can further impede their ability to function effectively in an already challenging work environment [5, 6]. Consequently, low morale and work-related stress have emerged as critical concerns within the profession [3, 7]. Despite these challenges, many midwives exhibit remarkable resilience, maintaining strong commitment to their roles and delivering essential care—a phenomenon closely tied to *work engagement* [7].

Work engagement, a pivotal concept in organizational psychology, refers to a positive, fulfilling work-related state characterized by vigor, dedication, and absorption [8, 9]. Extensive research demonstrates its association with greater work performance [10–12]. Studies across Europe, Asia, and North America have consistently linked high levels of work engagement to high work performance [10, 13–16]. Highly engaged workers exhibit greater productivity, motivation, and organizational loyalty [16]. Multiple factors influence work engagement, including leadership style, organizational culture, job resources, recognition, workload, and opportunities for growth [17, 18]. Among these, *organizational commitment*—an worker’s psychological attachment to their institution has been widely studied. Research on nurses and physicians in both developed and developing contexts, including Ghana, highlights its impact on performance [19–22]. However, few studies have examined this relationship among midwives, despite their unique role in maternal-newborn health [23].

In Ghana, midwives serve as the backbone of maternal and neonatal healthcare, providing antenatal, delivery, and postnatal services [3]. Despite health system improvements, they face persistent challenges such as staffing shortages, high patient volumes, and inadequate resources [24]. Additionally, midwives often report feeling undervalued compared to nurses, with limited involvement in decision-making and professional advancement; factors that undermine morale and engagement [24–26].

The implications of midwives’ performance extend beyond the workplace and into the broader realm of public health. Ghana’s maternal mortality (310 deaths per 100,000 live births) and neonatal mortality (25 deaths per 1,000 births) rates remain unacceptably high despite increased access to skilled birth attendance [27, 28]. These statistics underscore the urgent need to strengthen midwifery services by addressing workforce-related barriers. Therefore, investigating the mediating effect of work engagement on the relationship between organizational commitment and work performance is essential for designing targeted interventions. Such efforts are vital not only for achieving Sustainable Development Goal 3 (Good Health and Well-being) but also for safeguarding maternal and neonatal health outcomes. This study, therefore, examined this mediating relationship among Ghanaian midwives.

### Literature review and research hypotheses

#### Organizational commitment and work engagement

Organizational commitment and work engagement are two important constructs in organizational psychology that influence worker performance, job satisfaction, and overall workplace productivity [11]. Organizational commitment refers to workers’ psychological attachment to their organization, characterized by their willingness to remain with the organization and exert effort on its behalf [29, 30]. Meyer and Allen [30] conceptualized organizational commitment as a multidimensional construct comprising: Affective commitment (emotional attachment to the organization), Continuance commitment (retention based on perceived costs of leaving), and Normative commitment (a felt obligation to remain) [31].

Work engagement, on the other hand, is defined as a positive, fulfilling, work-related state of mind characterized by vigor, dedication, and absorption [32]. Although both concepts are related to workers’ motivation and retention, they differ in their underlying mechanisms. Organizational commitment is more about workers’ loyalty to the organization [33], whereas work engagement reflects workers’ emotional and cognitive investment in their work [34]. Research indicates that workers with high organizational commitment tend to show greater work engagement, driven by a stronger connection to their roles and responsibilities [18].

The relationship between organizational commitment and work engagement is grounded in the Social Exchange Theory (SET). According to SET, the link between organizational commitment and work engagement is rooted in the presence of high-quality social exchange relationships, which foster both a sense of loyalty and active engagement at work [35]. Receiving socio-emotional resources such as respect, recognition, job security, and fair treatment signals to workers that they are valued by the organization. This perception builds trust and evokes a sense of obligation to reciprocate, resulting in stronger organizational commitment [36] and increased motivation to engage in productive work behaviors [37]. So, when an organization consistently supports its workers through development opportunities and fosters a positive work culture, workers are more likely to feel emotionally bonded and loyal to the organization. This emotional attachment often translates into a deeper investment in their work roles [38–40]. Studies in the healthcare sector underscore the significance of these constructs. Organizational commitment has been shown to enhance the level of work engagement among healthcare professionals. For example, cross-sectional studies conducted by Cao et al. [41], Tang et al. [42], and Shdaifat et al. [43] reported that organizational commitment positively influences work engagement among nurses.

#### Work engagement and work performance

Work performance, in this context, encompasses both task performance (fulfilling core job responsibilities) and contextual performance (extra-role behaviors such as helping colleagues and volunteering for additional tasks) [44]. Work engagement and work performance are closely linked constructs that play a vital role in organizational success.

The relationship between work engagement and performance is well-explained by the Job Demands–Resources (JD-R) model. According to this model, every occupation has inherent risk factors associated with job demands and job resources [45]. Job demands refer to aspects of the job that require sustained physical or mental effort and are associated with certain physiological and psychological costs, such as workload, time pressure, and emotional demands [46]. In contrast, job resources are physical, psychological, social, or organizational elements that help workers achieve work goals, reduce job demands, or promote personal growth and development—examples include autonomy, performance feedback, supervisory support, and development opportunities [47].

The JD-R model posits that job resources are especially significant in fostering worker motivation, which in turn leads to greater work engagement and ultimately improved performance [48]. When workers have adequate job resources, they are more likely to experience high levels of engagement. They are typically energetic in the face of challenges, dedicated to their responsibilities, and deeply immersed in their tasks often to the point of losing track of time due to their absorption in the work [49]. This psychological state of engagement enhances overall work performance [50].

In healthcare settings, the influence of work engagement is particularly pronounced. High engagement levels among frontline healthcare workers including nurses are positively associated with crucial outcomes including quality of care, patient satisfaction, and patient safety [51]. High level of engagement is linked to fewer medical errors, greater compliance with safety protocols, and more empathetic interactions with patients. Collectively, these outcomes exemplify improved work performance in clinical environments [51].

#### Organizational commitment and work performance

Organizational commitment has been widely recognized as a key determinant of work performance. A substantial body of research consistently demonstrates that workers with strong organizational commitment tend to exhibit higher levels of work performance [52]. Commitment also means that they are more willing to put in extra effort and take responsibility for achieving organizational goals, which directly contributes to their performance [53, 54].

One of the most compelling explanations for the relationship between organizational commitment and work performance is offered by Social Identity Theory [55]. This theory posits that individuals derive part of their self-concept from their membership in social groups, including organizations [56]. When workers strongly identify with their organization, their self-esteem becomes closely tied to organizational outcomes. In this context, poor performance may be perceived as a threat to their self-identity, while good performance enhances their personal and professional image [57]. Such identification motivates workers to uphold a positive view of both themselves and their organization by consistently performing well [56]. The stronger the organizational commitment, the more likely workers are to take ownership of their responsibilities, foster a collaborative work environment, and engage in behaviors that reinforce the organization’s success [58]. This dynamic helps explain why highly committed workers often go “above and beyond” their formal job duties because underperformance would conflict with their self-concept and values [56, 57].

In clinical settings where the quality of performance directly impacts patient outcomes, organizational commitment becomes particularly critical. Committed healthcare workers are more likely to adhere to safety protocols, collaborate effectively with interdisciplinary teams, and demonstrate empathy in patient care, all of which enhance service delivery and health outcomes [59]. Empirical evidence across diverse health sectors supports this connection. For example, studies have shown that frontline healthcare workers, such as nurses, with strong organizational commitment exhibit significantly higher levels of work performance. These behaviors reflect not only professional competence but also a deep sense of purpose and loyalty to the organization [43, 60–63].

### Mediating effect of work engagement on the relationship between organizational commitment and work performance

Work engagement is increasingly recognized as a pivotal construct in understanding the linkage between organizational commitment and work performance [64]. The dynamic interplay among these three variables has drawn considerable scholarly interest, particularly regarding the mediating role of work engagement [12, 65]. A growing body of literature suggests that work engagement operates as a key psychological mechanism through which organizational commitment translates into enhanced work performance [11]. Research consistently indicates that committed workers are more likely to become engaged in their work, and this heightened engagement subsequently leads to superior performance outcomes [37, 66]. Theoretically, work engagement represents a motivational state that enables committed workers to channel their energy, focus, and professional identity into performance-enhancing behaviors [67].

The Job Demands–Resources (JD-R) model offers a useful framework for understanding this mediating role. According to the model, both personal and job-related resources foster work engagement, which in turn promotes higher work performance [68, 69]. In this framework, organizational commitment functions as a personal resource that energizes workers to invest themselves physically, cognitively, and emotionally in their work. This increased engagement, in turn, leads to enhanced performance [11]. Christian et al. [10] emphasized that work engagement serves as a crucial link between antecedents such as organizational commitment and work performance outcomes. Similarly, Agu [70] noted that the combination of commitment and engagement fosters a positive organizational climate that supports performance excellence.

Committed workers often experience a stronger sense of purpose and belonging, which enhances both their engagement and performance [11]. Cesário and Chambel [12] further explained that organizational commitment reflects a worker’s willingness to contribute meaningfully and exceed basic job expectations. Workers with high levels of commitment tend to demonstrate high engagement, which then drives performance [12]. Supporting this perspective, Yalabik et al. [71] found that work engagement not only mediates the relationship between organizational commitment and work performance but also reduces turnover intentions.

In healthcare settings, particularly among frontline professionals, the impact of this relationship is significant. Nurses with high levels of work engagement frequently report stronger organizational commitment and are shown to deliver better performance, which directly contributes to improved patient care [43, 72].

**Fig. 1:**
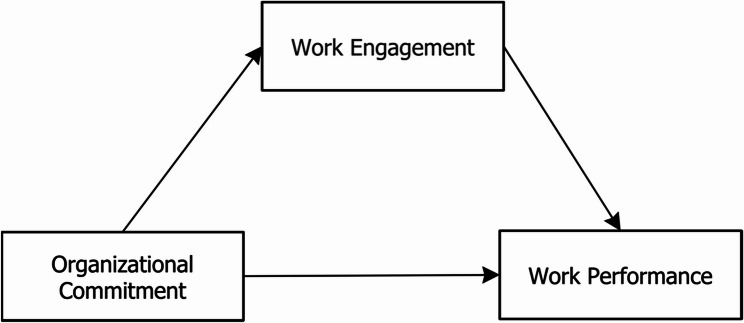
Conceptual model

Despite the extensive research on nurses, midwives, who play a distinct and vital role in maternal and child healthcare, have received far less attention in this area. Most international and African studies either focus exclusively on nurses or combine data in ways that obscure the unique experiences and contributions of midwives. Yet, as Freeney and Fellenz [73] emphasized, midwives’ work engagement is critical, as it directly influences their professional service delivery. Given the well-established mediating effect of work engagement in the commitment–performance relationship, this study specifically sought to examine this dynamic among midwives in Ghana. Building upon insights from existing literature, a conceptual model was developed to guide this study, as shown in Fig. 1. The model proposes the following hypothetical relationships:

- Hypothesis 1: Midwives’ organizational commitment would affect their work engagement.
- Hypothesis 2: Midwives’ work engagement would affect their work performance.
- Hypothesis 3: Midwives’ organizational commitment would affect their work performance.
- Hypothesis 4: Midwives’ work engagement would mediate the relationship between their organizational commitment and work performance.

## Materials and methods

### Study design

This study employed an analytical cross-sectional design to examine the mediating effect of work engagement on the relationship between organizational commitment and work performance among midwives in Ghana. The study was conducted over a three-month period (January-March 2025).

### Study setting and population

The study was conducted in three hospitals in the Tamale Metropolis, Northern Ghana. These hospitals are equipped with functional wards, laboratories, essential units, and provide round-the-clock healthcare services to people in Tamale and beyond. The total number of midwives across the three hospitals was 552 (Hospital A = 305, Hospital B = 130, and Hospital C = 117). The study population comprised registered midwives working at the three hospitals, regardless of rank or work experience.

### Sample size and sampling method

The sample size was calculated using Yamane [74] formula for finite populations:

$$\:n=\frac{N}{1+N{\left(e\right)}^{2}}$$

Where:

- *n =* required sample size,
- *N* = total population (552),
- *e =* margin of error (0.05).

The initial calculation yielded 231 participants; a 10% buffer for attrition resulted in a final sample of 254. A stratified random sampling method was employed, grouping hospitals based on the number of midwives. The sample was proportionally distributed as follows: Hospital A (305 midwives): 140 participants, Hospital B (130 midwives): 60 participants, and Hospital C (117 midwives): 54 participants. Simple random sampling was used to select participants from each hospital. The list of midwives in each ward was obtained and entered into SAS JMP Professional Statistical Software, where the random sampling function was applied to randomly select participants.

### Measures

#### Organizational commitment

The Organizational Commitment Scale developed and validated by Allen and Meyer [75], consisting of 18 items, was used to assess midwives’ organizational commitment across three dimensions: affective commitment, continuance commitment, and normative commitment. Each item was rated on a 5-point Likert scale (1 = “Strongly disagree” to 5 = “Strongly agree”), with reverse scoring applied to negatively worded items. The total score ranged from 1 to 5, with higher scores indicating greater organizational commitment. Previous studies have reported good internal consistency for the overall scale, with Cronbach’s alpha values of 0.88 [76] and 0.81 [59].

### Work engagement

The Utrecht Work Engagement Scale (UWES-9), developed by Schaufeli et al. [77], was used to measure midwives’ work engagement. This 9-item scale comprises three dimensions: vigor, dedication, and absorption. Responses were recorded on a 7-point frequency scale (0 = “Never” to 6 = “Always”). The total score was calculated as the average across all items, ranging from 0 to 6, with higher scores reflecting greater work engagement. The scale has shown high internal consistency in prior studies, with reported Cronbach’s alpha values of 0.93 [78] and 0.95 [79].

### Work performance

Work performance was assessed using the 18-item Individual Work Performance Questionnaire (IWPQ) developed by Koopmans et al. [80]. The questionnaire measures three dimensions: task performance, contextual performance, and counterproductive work behavior. Task and contextual performance items were rated on a 5-point scale (0 = “Seldom” to 4 = “Always”), while counterproductive work behavior items used a 5-point scale (0 = “Never” to 4 = “Often”). A mean score was computed for the overall scale, with higher scores indicating better work performance. Previous research has reported acceptable reliability for the scale, with Cronbach’s alpha values of 0.88 [81] and 0.86 [13].

### Validity and reliability

In this study, the work engagement, work performance, and organizational commitment scales were adapted and validated within the Ghanaian context to ensure cultural relevance and psychometric robustness. Prior to the main data collection, the adapted instruments were pilot-tested among a sample of 50 midwives to assess clarity, appropriateness, and preliminary reliability. The pilot testing revealed that all items were well understood and contextually appropriate; therefore, no revisions were necessary.

Following the pilot, the validity and reliability of the scales were carefully evaluated using multiple statistical metrics. Convergent validity was assessed using the Average Variance Extracted (AVE), which reflects the amount of variance captured by a construct in relation to the variance due to measurement error. An AVE value of 0.50 or higher is generally considered acceptable, indicating that the construct explains at least 50% of the variance in its items [82]. In this study, all scales met this criterion: work engagement (AVE = 0.64), work performance (AVE = 0.67), and organizational commitment (AVE = 0.61), demonstrating adequate convergent validity. In addition, composite reliability (CR) was also examined to assess the internal consistency of the constructs, with a recommended threshold of 0.70 or above indicating good reliability [83]. The CR values in this study were satisfactory across all scales: 0.89 for work engagement, 0.87 for work performance, and 0.80 for organizational commitment, suggesting that the items reliably measure their underlying constructs. Together, these findings confirm that the adapted scales demonstrate satisfactory validity and reliability, making them appropriate for use in the Ghanaian context.

### Data collection

The data collection process commenced after receiving formal approval from the management of the three participating hospitals. All participants provided both verbal and written informed consent following a comprehensive briefing about the study’s purpose, confidentiality protections, and voluntary nature of participation. The researchers distributed the questionnaires to the participants and supervised their completion. Participants were allotted 10–15 min to complete the questionnaires, with researchers available to ensure accurate responses. Each completed questionnaire was checked for missing or incomplete responses. To ensure privacy, questionnaires were sealed in envelopes labeled with unique identification codes to prevent personal identification. These envelopes were securely stored throughout the study to maintain confidentiality.

### Statistical analysis

Data entry and cleaning were performed using SPSS Statistics version 27. Descriptive statistics summarized sociodemographic characteristics and study variables. Pearson correlation analysis examined relationships between organizational commitment, work engagement, and work performance. Mediation analysis was conducted using SPSS AMOS version 24 with 5,000 bootstrap resamples to assess both direct and indirect effects of organizational commitment on work performance, with work engagement as the mediator. Bootstrapping is a non-parametric resampling method commonly used in mediation analysis to test the significance of indirect effects without assuming normality of the sampling distribution. It provides more accurate confidence intervals, particularly for smaller samples or non-normal data. A resample size of 5,000 is generally recommended to ensure stable estimates and sufficient power [84]. Model fit was assessed using several fit indices in AMOS, including the relative Chi-square (CMIN/DF), Comparative Fit Index (CFI), Tucker-Lewis Index (TLI), Root Mean Square Error of Approximation (RMSEA), and Standardized Root Mean Square Residual (SRMR). Acceptable model fit was determined using the following commonly recommended cutoffs: CMIN/DF ≤ 5, CFI ≥ 0.90, TLI ≥ 0.90, RMSEA ≤ 0.08, and SRMR ≤ 0.08 [85, 86]. All statistical test assumptions were verified prior to analysis. A p-value < 0.05 was considered statistically significant.

### Ethical consideration

Ethical approval was obtained from the Committee on Human Research, Publications, and Ethics at Kwame Nkrumah University of Science and Technology (CHRPE/AP/013/24). Administrative permission was secured from all three participating hospitals. Written and verbal informed consent was obtained from all participants prior to data collection, with only eligible, consenting individuals included in the study. Participants were reminded of their right to withdraw at any time without consequence. Confidentiality was strictly maintained, with no personal identifiers linked to study data.

## Results

### Sociodemographic characteristics

Table 1 displays participant sociodemographic characteristics. Most participants were aged 30–39 years (54.3%) and female (96.9%). The majority were married (72.0%), while 37.0% had 1–2 children. Over half held a bachelor’s degree or higher (61.4%), and slightly more than half (50.4%) had less than five years of work experience.

**Table 1:** Sociodemographic characteristics of the participants

Variable	Frequency	Percentage
Age (years)		
20–29	72	28.3
30–39	138	54.3
40–49	44	17.3
Gender		
Male	8	3.1
Female	246	96.9
Marital status		
Single	71	28.0
Married	183	72.0
Number of children		
0	75	29.5
1–2	94	37.0
>2	85	33.5
Education status		
Bachelor’s degree and higher	156	61.4
Diploma	98	38.6
Hospital type		
Primary hospital	114	44.9
Tertiary hospital	140	55.1
Work experience (years)		
<5	128	50.4
5–10	109	42.9
>10	17	6.7
Rank		
Staff midwife	118	46.5
Senior midwife	115	45.3
Principal midwife	21	8.3

### Descriptive statistics

Table 2 presents the descriptive statistics for the study variables. Organizational commitment showed a mean score of 3.05 (SD = 0.54) with a median of 3.06. Work engagement demonstrated a mean of 2.41 (SD = 0.96) and median of 2.56. Work performance recorded the lowest mean score at 2.01 (SD = 0.67), with a median of 2.06.

**Table 2:** Descriptive statistics of study variables

		95% CI					
	Mean	Lower	Upper	Median	SD	Minimum	Maximum
Organizational commitment	3.05	2.98	3.12	3.06	0.542	1.17	4.44
Work engagement	2.41	2.29	2.53	2.56	0.963	0	4
Work performance	2.01	1.93	2.09	2.06	0.672	0	4

“CI” Denotes confidence interval, “SD” Denotes standard deviation

### Correlation analysis

As presented in Table 3, the correlation analysis results supported all three hypotheses. Hypothesis 1, which posited that midwives’ organizational commitment would affect their work engagement, was accepted, as a significant positive correlation was observed (*r* = 0.125, *p* < 0.05). Hypothesis 2, which suggested that midwives’ work engagement would affect their work performance, was supported by a strong, significant positive correlation (*r* = 0.662, *p* < 0.05). Hypothesis 3, which proposed that midwives’ organizational commitment would affect their work performance, was also accepted, with a significant positive correlation (*r* = 0.166, *p* < 0.05).

**Table 3:** Correlation between organizational commitment, work engagement, and work performance

	Organizational commitment	Work engagement	Work performance
Organizational commitment	—		
Work engagement	0.125*	—	
Work performance	0.166*	0.662*	—

*Statistically significant at *p* < 0.05

### Mediation analysis

**Fig. 2:**
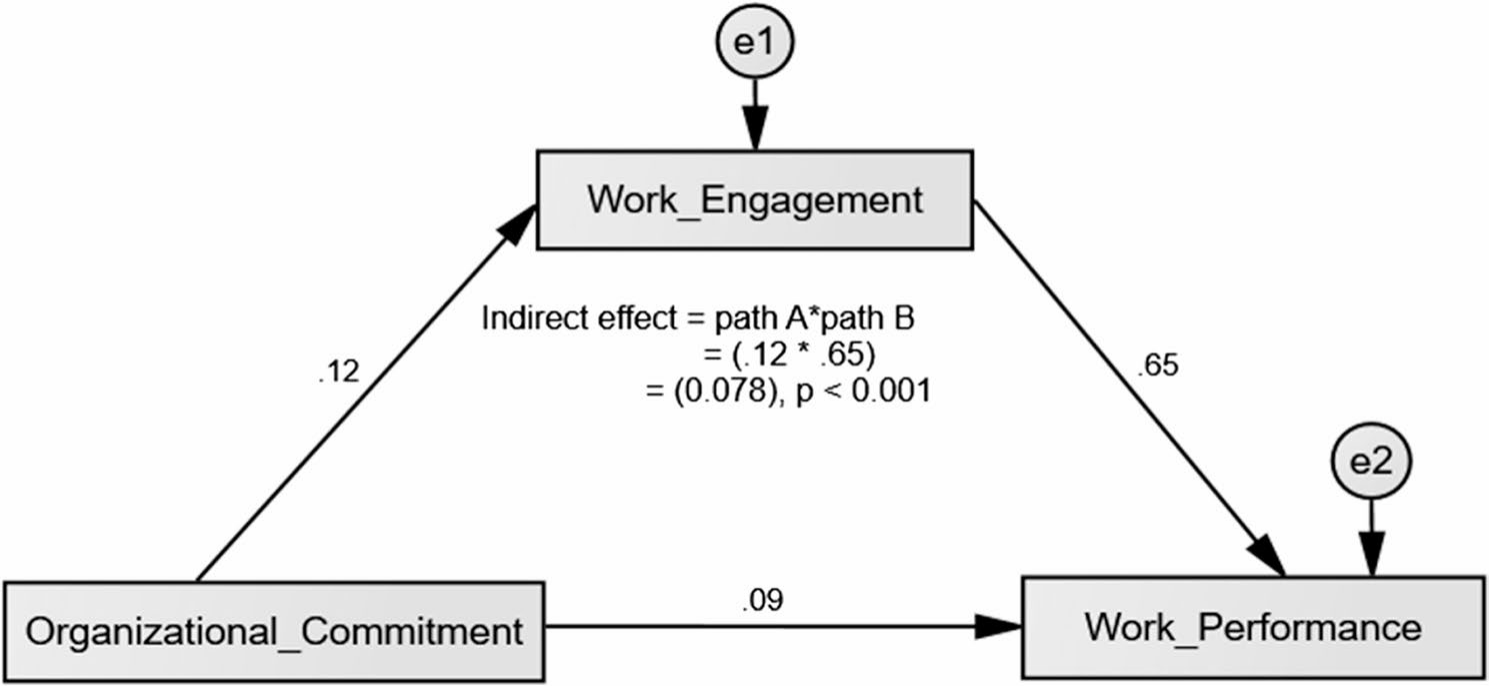
Simple mediation model of the study variables

As shown in Table 4, the simple mediation analysis examined work engagement’s mediating effect between organizational commitment and work performance. The structural equation model demonstrated acceptable fit to the data: CMIN/DF = 2.81, CFI = 0.954, TLI = 0.931, RMSEA = 0.055, and SRMR = 0.043. These values met the recommended thresholds for acceptable model fit. Results revealed that organizational commitment significantly predicted work engagement (β = 0.124, SE = 0.111, *p* = 0.046), which in turn significantly predicted work performance (β = 0.651, SE = 0.033, *p* < 0.001). While the direct effect of organizational commitment on work performance was non-significant (β = 0.085, SE = 0.090, *p* = 0.072), the indirect effect through work engagement was significant (β = 0.078, SE = 0.061, *p* < 0.001), demonstrating full mediation (Fig. 2). These findings support Hypothesis 4, confirming that midwives’ work engagement would mediate the relationship between their organizational commitment and work performance.

**Table 4:** Direct and indirect effects of organizational commitment on work performance through work engagement among midwives

Model pathway	Unstandardized Coefficient	Std. Error	Critical Ratio	Standardized Coefficient	Sig.
	(B)			(Beta)	
Direct Effect					
Work Engagement **<---**Organizational Commitment	0.221	0.111	1.997	0.124	0.046*
Work Performance **<---**Work Engagement	0.454	0.033	13.788	0.651	< 0.001*
Work Performance **<---**Organizational Commitment	0.105	0.090	1.801	0.085	0.072
Indirect Effect					
Work Performance **<---** Work Engagement **<---** Organizational Commitment	0.101	0.061	0.055	0.078	< 0.001*

*Statistically significant at *p* < 0.05

### Common method bias assessment

Common method bias (CMB) was assessed to ensure that variance attributable to the measurement method, rather than the constructs of interest, did not unduly influence the results. A widely used approach for this purpose is Harman’s single-factor test, which involves conducting an exploratory factor analysis (EFA) on all measurement items using unrotated principal component extraction. The rationale is that if a single factor emerges, or if one factor accounts for a majority of the variance (typically 50% or more), it may suggest the presence of common method variance [87]. In this study, all items from the work engagement, work performance, and organizational commitment scales were included in the analysis. The results revealed that the first factor accounted for only 22.3% of the total variance, well below the 50% cutoff. This indicates that common method bias is unlikely to pose a serious threat to the validity of the study’s findings.

## Discussion

This study examined the mediating effect of work engagement on the relationship between organizational commitment and work performance among midwives. The study’s findings contribute to the existing body of literature by validating and extending theoretical frameworks related to worker engagement and performance, particularly in healthcare settings within low- and middle-income countries. By connecting these results to earlier research, the study offers fresh insights into how organizational commitment and work engagement influence performance in the context of maternal healthcare.

The first hypothesis, which proposed that midwives’ organizational commitment would affect their work engagement, was supported. This aligns with previous studies by Khalid and Khalid [51], Cesário and Chambel [12], Arcadio et al. [88], which reported that organizational commitment fosters higher levels of engagement. These findings reinforce the idea that when workers feel emotionally attached to and identify with their organization, they are more likely to invest themselves enthusiastically in their work. However, this study extends the literature by confirming this relationship specifically among midwives in the Ghanaian healthcare system where persistent challenges such as understaffing and limited resources often impact morale [7]. Despite working under challenging conditions, many midwives in Ghana demonstrate unwavering commitment to their roles. This strong sense of professional dedication often sustains high levels of engagement, even in the face of limited material resources. Their enduring sense of duty not only fuels their performance but also strengthens their bond with the healthcare system [3]. The study highlights organizational commitment not merely as a contributing factor but as a foundational driver of work engagement among midwives. Nonetheless, not all studies support this association. For example, Jackson [89] found no significant relationship between organizational commitment and engagement, suggesting that the strength of this link may vary based on contextual factors such as organizational culture, leadership style, and work conditions. This variability underscores the need for more context-specific research.

The second hypothesis, which proposed that midwives’ work engagement would affect their work performance, was also supported by the findings. This is consistent with prior research by Sekha [15], Sittar [90], and Mundhra and Pramanik [91], which reported a positive relationship between engagement and performance. In this study, high levels of engagement translated into improved performance among midwives—an outcome that is essential for delivering high-quality maternal care. This adds to the literature by showing that enhancing work engagement can have broader public health benefits, particularly in improving maternal and neonatal outcomes [92]. While some scholars have argued that variables like creativity or job satisfaction mediate the engagement-performance relationship [93, 94], the current study demonstrates that engagement alone can be a powerful independent predictor of performance, especially in resource-constrained environments. This finding suggests that promoting a strong sense of purpose and teamwork among midwives may significantly boost both engagement and performance even without structural reforms.

The third hypothesis, which posited that midwives’ organizational commitment would affect their work performance, was supported by the data. This result corroborates previous studies by Dinc [95], Suharto et al. [96], Krishnanathan and Mangaleswaran [97], Beigi and Lajevardi [98], and Cobbinah et al. [99], which have established that organizational commitment is a significant predictor of work performance. This study adds depth by confirming the role of organizational commitment in improving work performance specifically among midwives [100]. Contextually, in Ghana’s healthcare system, performance often depends not only on skills but also on the individual’s sense of responsibility to their patients and communities. A committed midwife does not just perform tasks but they ensure mothers are emotionally supported, procedures are followed despite challenges, and critical decisions are made with compassion. These behaviors aren’t always in the manual but they are what make the difference. Thus, commitment in this setting is more than a psychological bond; it is a moral compass that influences how work is done. Nonetheless, it is important to acknowledge conflicting findings in other studies, such as Tawiah [101] and James [102], where organizational commitment did not affect work performance. These divergent results could be due to variations in organizational culture or work environment.

The fourth hypothesis, which proposed that midwives’ work engagement would mediate the relationship between their organizational commitment and work performance, was supported through a confirmed full mediation. This aligns with findings by Suharto and Suprapto [103], who found that organizational commitment influences performance indirectly through engagement. Studies by Yalabik et al. [71] and Cesário and Chambel [12] similarly emphasized that committed workers tend to show higher engagement, which then drives performance. In the context of midwifery in Ghana, this mediation makes practical sense. Many midwives report feeling more energized and dedicated when they receive emotional support from supervisors or feel trusted in their roles. The stronger their bond with the institution, the more likely they are to be fully engaged, proactive, and resilient. In this way, engagement becomes the engine that converts commitment into performance. As one might put it metaphorically, commitment is the match, but engagement is the fuel [104, 105]. This study challenges overly simplistic interpretations of the commitment-performance link. While some research has suggested that commitment alone is sufficient to enhance performance [95–97], the present study shows that engagement is a critical psychological bridge between the two. These insights carry important implications for healthcare administrators aiming to enhance service delivery in maternal healthcare. To drive sustainable improvements in performance, policies and programs should simultaneously strengthen both organizational commitment and work engagement. Importantly, these initiatives must be tailored to the Ghanaian context. Interventions such as leadership development, peer-support systems, and culturally sensitive recognition programs are more likely to succeed than externally imposed models. Midwives must not only be well-trained but also feel seen, respected, and valued. That is how commitment transforms into energy and energy into meaningful impact. By fostering both commitment and engagement, healthcare organizations can enhance midwives’ well-being, elevate work performance, and ultimately improve maternal and neonatal outcomes.

### Limitations

Despite the rigorous methodology, this study has several limitations. First, the cross-sectional design restricts the ability to infer causality between organizational commitment, work engagement, and work performance. Second, the study relied solely on self-reported measures, which may be subject to social desirability and recall biases, potentially inflating the relationships among variables. Third, although the scales were adapted and validated in the Ghanaian context, cultural nuances specific to the Tamale Metropolis or unique experiences of midwives in these settings might not be fully captured by the standardized instruments. Fourth, while stratified random sampling was employed, limiting the study to only three hospitals in one metropolis may constrain the generalizability of the findings to other healthcare settings or regions within Ghana. Lastly, the study’s sample was overwhelmingly female. However, this gender imbalance limits the generalizability of the findings to male midwives, whose experiences and perspectives on work engagement, organizational commitment, and performance may differ.

## Conclusion

This study provides valuable insights into the relationships among organizational commitment, work engagement, and work performance in midwives. Our findings demonstrate that enhancing midwives’ organizational commitment significantly improves both their work engagement and performance. The results further reveal that increased work engagement leads to substantially better performance, with work engagement serving as a full mediator between organizational commitment and work performance. These results suggest that organizational commitment boosts work performance primarily through its positive effect on work engagement, highlighting the crucial role of engagement in translating commitment into improved performance.

### Practical implications

The Ministry of Health (MoH) in Ghana should incorporate organizational commitment and work engagement strategies into national healthcare policies, particularly by developing frameworks that recognize work engagement’s mediating role in performance enhancement. Healthcare institutions should cultivate supportive work environments that value midwives’ contributions through incentive programs, open communication channels, and continuous professional development opportunities. To specifically enhance work engagement among midwives in Ghana, healthcare facilities should implement regular recognition and reward programs that acknowledge both individual and team achievements, such as “Midwife of the Month” awards or performance-based bonuses. Institutions should also establish structured mentorship and peer support systems to foster professional growth and emotional support. Additionally, introducing flexible work schedules where possible and promoting participatory decision-making can empower midwives and increase their sense of ownership and dedication to their roles. Ensuring adequate staffing levels to reduce workload pressure and prevent burnout is another critical practice that can sustain vigor and absorption at work. These measures will help maintain high engagement levels and motivation among midwifery staff, ultimately improving healthcare service delivery.

Beyond the clinical setting, these findings have important educational implications. Training institutions for midwives should integrate content on organizational behavior, emotional intelligence, and engagement strategies into their curricula to prepare future professionals for dynamic healthcare environments. Embedding these topics early in professional education could foster long-term commitment and resilience in the workforce.

From a policy perspective, workforce planning strategies should be informed by the evidence that engaged and committed midwives perform better. National staffing models and retention policies should therefore emphasize job enrichment, recognition systems, and opportunities for role autonomy. Specifically, policy makers could mandate periodic assessments of work engagement levels as part of routine human resource audits across public hospitals.

While this study clarifies work engagement’s mediating role, future research should explore additional mediators and moderators in the commitment-performance relationship. Longitudinal studies would particularly strengthen our understanding of these causal relationships over time. Furthermore, practical tools such as engagement assessment dashboards or commitment-performance tracking systems should be developed and piloted within hospitals to help administrators make data-driven decisions. We encourage continued investigation in this area to develop targeted interventions that enhance midwives’ performance, recommending that stakeholders support such evidence-based research to improve Ghana’s healthcare system.

## Data Availability

The data used and/or analyzed in this research are available from the corresponding author upon reasonable request, provided that participants’ confidentiality is maintained.
